# Physical activity for chronic back pain: qualitative interview study with patients and GPs in German primary care

**DOI:** 10.3399/BJGP.2022.0215

**Published:** 2023-04-04

**Authors:** Nicole Lindner, Nele Kornder, Julia Heisig, Veronika van der Wardt, Annika Viniol

**Affiliations:** Department of general practice/family medicine, University of Marburg, Marburg, Germany.; Department of general practice/family medicine, University of Marburg, Marburg, Germany.; Department of general practice/family medicine, University of Marburg, Marburg, Germany.; Department of general practice/family medicine, University of Marburg, Marburg, Germany.; Department of general practice/family medicine, University of Marburg, Marburg, Germany.

**Keywords:** back pain, exercise, physician–patient relationships, primary health care, qualitative research, treatment adherence and compliance

## Abstract

**Background:**

Chronic back pain (CBP) is common among patients in primary care and is associated with significant personal and socioeconomic burden. Research has shown that physical activity (PA) is one of the most effective therapies to reduce pain; however, for GPs it remains challenging to advise and encourage individuals with CBP to exercise regularly.

**Aim:**

To provide insight into the views and experiences of PA in individuals with CBP, along with those of GPs, and to reveal the facilitators and barriers to engaging in, and maintaining, PA.

**Design and setting:**

Qualitative semi-structured interviews with individuals with CBP and GPs recruited via the local research practice network (Famprax) in Hessen, western-central Germany between June and December 2021.

**Method:**

Interviews were coded separately by consensus and analysed thematically. Findings of the two groups (GPs and patients with CBP) were compared and summarised.

**Results:**

A total of 14 patients (*n* = 9 females and *n* = 5 males) and 12 GPs (*n* = 5 females and *n* = 7 males) were interviewed. Opinions and experiences of PA in individuals with CBP were similar both within and across the GP and patient groups. Interviewees expressed their views on internal and external barriers to PA, and provided strategies to address these barriers and concrete recommendations to increase PA. This study revealed a conflicting doctor–patient relationship ranging from paternalistic, to partnership based, to service provision, which could lead to negative perceptions on both sides, such as frustration and stigma.

**Conclusion:**

To the best of the authors’ knowledge, this is the first qualitative study exploring opinion and experience of PA in individuals with CBP and GPs in parallel. This study reveals a complex doctor– patient relationship and provides an important insight to motivation for, and adherence to, PA in individuals with CBP.

## INTRODUCTION

Chronic back pain (CBP) is common in patients in primary care and is associated with significant personal and socioeconomic burden. It is one of the leading causes contributing to disability worldwide^1^ and results in enormous direct health care and lost-productivity costs.^2^^,^^3^

There is a robust body of evidence showing that various types of physical activity (PA) lead to an improvement in back pain,^2^^,^^4^ and numerous clinical guidelines recommend PA as the primary treatment for CBP.^5^^–^^7^ However, adherence to any health intervention is difficult, even more so in individuals with chronic conditions.^8^ In this context, Caspersen *et al* defined PA as any bodily movement that results in energy expenditure, and exercise as a planned structured and repetitive subset of PA to improve physical fitness.^9^

In the general population PA activity is insufficient: one in two females and one-third of males in England do not achieve the targets of the UK guidelines on PA.^10^^,^^11^ Individuals with CBP have insufficient levels of PA independent of their pain-related disability.^12^^,^^13^ Poor adherence to PA recommendations limits its potential long-term effectiveness. Studies have explored various strategies, including goal setting and use of self-monitoring techniques, to improve adherence to PA.^14^ However, research has shown that adherence is influenced by several interdependent factors, such as patients’ characteristics and clinical setting, and even fluctuates over time.^15^^,^^16^

Qualitative studies show the importance of the role of health professionals and their impact in promoting PA in patients with CBP, and GPs tend to view it as their duty to promote PA.^17^^,^^18^ Current approaches for promoting PA in primary care are ineffective and it remains challenging for GPs to advise individuals with CBP to exercise regularly.^14^ Therefore, the aim of the present study was to explore individuals with CBP and their experience of PA and how, in their own and the GP‘s perspective, patients could be supported to increase their PA levels as part of their CBP therapy. The objective was also to understand the barriers and facilitators of PA and maintaining higher levels of PA.

## METHOD

### Study design

A semi-structured interview study was conducted with individuals with CBP and GPs, requesting that they explore their views and experience of PA for CBP (see Supplementary Figure S1). The study aimed to explore how patients could be supported to increase PA levels as part of their CBP therapy. Based on these findings, the researchers plan to develop an intervention (digital consultation tool) to increase PA in patients with CBP. The researchers chose this semi-structured interview approach to reveal the barriers and facilitators to PA for patients with CBP managed in primary care. Following an explanation of the nature and possible consequences of the study, informed written consent was obtained from all participants. The protocol followed the tenets of the Declaration of Helsinki.^19^ Members of a patient advisory board supported all phases of the project, for example, the development of a topic guide, while a physiotherapist supported the analysis. The research team is multidisciplinary (with backgrounds in medicine, psychology, and biology) and all have experience in qualitative research. All researchers reflected on their personal experience of PA and, where relevant, their experience of working as a GP. The study process underwent an internal peer review by the entire working group and an external peer review through a presentation at the German GP Congress.

**Table:** How this fits in

Physical activity (PA) is one of the most effective therapies for chronic back pain (CBP); however, it remains challenging to advise and encourage individuals with chronic pain to exercise regularly. As yet, quantitative and qualitative research has mainly focused on patients’ and doctors’ perspectives separately without taking the doctor–patient relationship into consideration. This study reveals the perceptions, barriers, and motivators of PA in the treatment of CBP, and the influence of conversations between patients and GPs around PA on the doctor–patient relationship.

### Study sample

The interview study participants were divided into two groups: GPs; and patients with CBP. GPs and patients were recruited via the local research practice network (Famprax) in the state of Hessen, western-central Germany. Inclusion criteria for GPs were that they were currently practising and that there was a computer in the consulting room. Inclusion criteria for patients were: having CBP; a minimum of three contacts with the GP because of CBP in the last 6 months; and being aged >18 years. An exclusion criterion was severe cognitive impairment. Participants of different socioeconomic backgrounds, ethnicities, age, and those from rural and urban settings were recruited via purposive sampling. Initially, patients were recruited by their known individual GP. To search for divergent data, recruitment was extended beyond the research practices after performing the first interviews. Additionally, the researchers advertised the study in practices and supermarkets using posters. Potential participants contacted the research group via mail/telephone and were provided with written and verbal information on the study. Recruitment was terminated after no new themes emerged and sufficient data had been collected to answer the study question.

### Data collection

Two semi-structured interview guides were developed by the study group after an extensive literature review in order to explore views on, and experience with, PA in individuals with CBP and GPs advising them. The interview guides were tailored after feedback from the patient advisory board and after performing the first interviews. In addition, the researchers discussed the first results within the study group to tailor the interview guides. An overview of main topics and corresponding sample questions are presented in Box 1. Demographic data were collected using a questionnaire. Interviews were conducted between June and December 2021. Due to COVID-19 restrictions, a combination of in-person, telephone, and video interviews was performed. Patient interviews were conducted at their homes and GP interviews at their practices. All interviews and analyses were held in German as all participants could speak and understand German.

**Box 1.** Interview guides. Overview of main topics and corresponding sample questions discussed in the interviews

**Interview guide for patients**	**Interview guide for GPs**
**Main topic:** patients prior clinical and personal history **Sample question** *‘Firstly, I would be interested in knowing for how long you have been experiencing back pain.’*	**Main topic:** experience on conversation on PA with patients with CBP **Sample question** *‘Firstly, I am interested in your experiences of the extent to which PA and exercise is a subject when you are consulting patients with chronic back pain.’*
**Main topic:** previous consultation with GP **Sample question** *‘Could you think back to the situation in which you last talked to your doctor about your back pain? What did you talk about?’*	**Main topic:** challenges in advising patients with CBP on PA **Sample question** *‘What challenges do you face in counselling patients with back pain towards more physical activity?’*
**Main topic:** previous therapy and its impact on pain **Sample question** *‘What do you do to treat your pain?’*	**Main topic:** supporting patients with CBP with PA in general **Sample question** *‘In your opinion, how could patients be supported to become more physically active?’*
**Main topic:** feelings on rest to treat back pain **Sample question** *‘“You must rest a hurting back.” What do you think about this statement?’*	**Main topic:** supporting patients with CBP with PA in primary care **Sample question** *‘How can you help the patients as their doctor?’*
**Main topic:** feelings on PA **Sample question** *‘Research says that movement is good against chronic pain. What do you think about that?*	**Main topic:** views on visualisation of impact of PA on pain **Sample question** *‘In our project we tried finding a good way to visualise the change of pain. We would love to hear your opinion on the different possibilities we have come up with.’*
**Main topic:** motivators and barriers to regular PA **Sample question** *‘What do you need to be able to exercise more?’*	
**Main topic:** views on visualisation of impact of PA on pain **Sample question** *‘In our project we tried finding a good way to visualise the change of pain. We would love to hear your opinion on the different possibilities we have come up with.’*	

*Two different interview guides were used for patients and GPs. The interview guide was tailored after having performed the first interviews and following discussion of the first results with the whole study group. CBP = chronic back pain. PA = physical activity.*

### Data analysis

Interviews were audiorecorded and transcribed verbatim. Quotes were translated into English by an external translator. Transcripts and translated quotes were double checked. Additionally, field notes were taken. Data were anonymised and all participants received pseudonyms (fictitious names according to the recommendation on pseudonymisation).^20^ Data were managed in MAXQDA (2022) and coded by consensus following the method of Braun and Clarke using a deductive– inductive approach; with interview questions supporting theme development (deductive) but participant answers allowing new themes to emerge (inductive).^21^^,^^22^

As a first step, the researchers familiarised themselves with the interviews by listening to the audio files of the interviews, reading the transcripts, and adding comments. In the second step, text passages that could be relevant to the research question were identified and codes were assigned to them.

In the third step, these codes were grouped into superordinate themes that were clearly distinguishable from each other and could be substantiated by codes. The final coding frame was reviewed in a sample of four interviews. Emerging themes were discussed with the study group and, initially, separate thematic maps were created with FreeMind (version 1.0.1) for patient and doctor interviews (see Supplementary Figures S2a and S2b). After further analysis of results within the study group, thematic maps were optimised in an iterative process. Themes arising among doctor and patient interviews were compared with each other. A complete study overview can be found in Supplementary Appendix S1.

## RESULTS

A total of 14 patients (*n* = 9 females and *n* = 5 males) and 12 GPs (*n* = 5 females and *n* = 7 males) were interviewed. Most of the interviews were completed via telephone (*n* = 17 telephone, *n* = 5 in person, and *n* = 4video conference). Interviews lasted between 15 and 52 minutes. Patient age ranged from 30 to 68 years, and that of GPs from 31 to 62 years. Characteristics of interview partners (GPs and patients) are presented in Table 1. Two patients who initially agreed to participate could not be interviewed because of failure to reach them again. In one interview, recording failed and detailed notes were used for coding.

**Table 1.** Characteristics of interview partners

**GPs characteristics and demographics (*n* = 12)**				**Patient characteristics (*n* = 14)**				

**GP^a^**	**Age, years**	**Time working as GP, years**	**Setting**	**Patient^a^**	**Age, years**	**Physical activity in daily life**	**Frequency of GP consultation, times per year^b^**	**Pain, mean^c^**
Anke, F,	45	6–15	Rural	Inge, F,	65	Intermediate	1–4	7
Hannah, F,	35	<5	Rural	Christian, M,	59	Intermediate	1–4	4
Riccardo, M,	62	>15	Rural	Cordula, F,	47	High	5–12	3.5
Melanie, F,	61	>15	Urban	Anastasia, F,	NG	NG	NG	NG
Rolf, M,	53	>15	Rural	Dmytro, M,	42	High	1–4	5.5
Matthias, M,	NG	NG	Rural	Barbara, F,	58	Low	>12	6.5
Karl, M,	60	6–15	Rural	Anna, F,	42	High	>12	7
Felix, M,	46	6–15	Urban	Harald, M,	30	High	5–12	8.5
Lukas, M,	42	NG	Urban	Birgitt, F,	51	High	1–4	4.5
Lena, F,	42	<5	Urban	Susanne, F,	68	Intermediate	5–12	8
Carla, F,	31	<5	Urban	Hans, M,	44	High	5–12	7
Maximilan, M,	40	6–15	Urban	Jana, F,	46	Intermediate	5–12	5
				Louisa, F,	39	Intermediate	1–4	3.5
				Karsten, M,	68	Low	1–4	2

*Detailed data for one patient is missing.*

a *All participants received pseudonyms: fictitious names according to the recommendation on pseudonymisation.^20^*

b *Consultations for chronic back pain.*

c *Assessed with a visual analogue numerical rating scale by asking ‘Indicate the severity of your pain below. Please tick on the scale below how much pain you feel on average’. NG = not given.*

The following themes emerged in the GP and patient groups:

1. positive attitude towards PA with regard to pain;
2. 2a. internal and 2b. external barriers affecting PA;
3. different features and qualities of supporting strategies and concrete ideas to increase adherence to PA;
4. influence on the doctor–patient relationship; and
5. some negative emotions from both GPs and patients.

**Figure 1.**
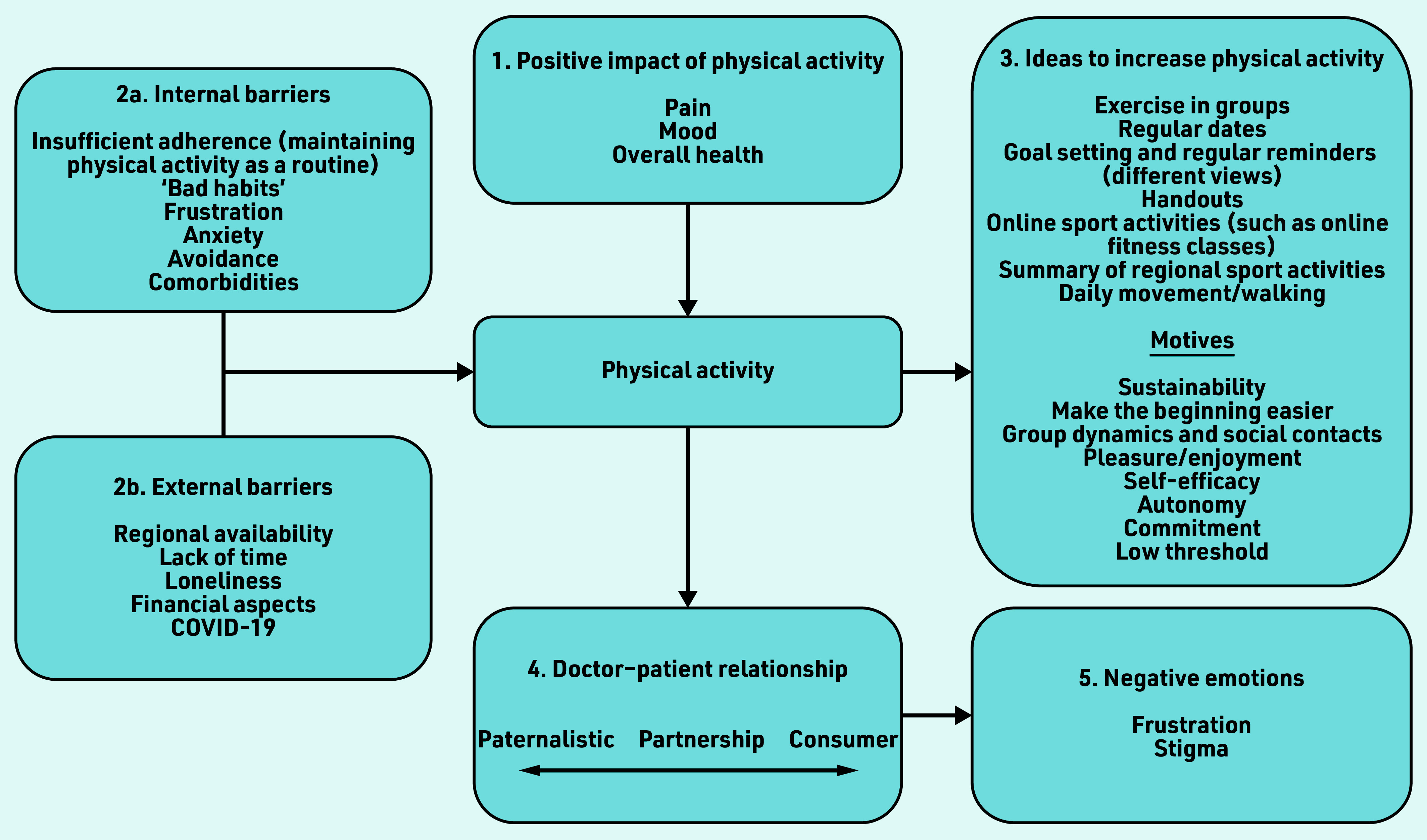
*Overview of factors influencing physical activity (PA) as expressed by patients and GPs. Themes were nearly identical in the GP and patient groups. Interview partners expressed 1. a positive impact of PA on pain; 2a. internal and 2b. external barriers leading to inactivity; 3. ideas to increase PA and motives for recommending them; 4. the doctor– patient relationship as a potential source of conflict, the implemented model ranged from paternalistic to partnership-based, to a consumer model; leading to 5. negative emotions on the side of GPs and patients.*

Figure 1 provides an overview of the important factors influencing PA. Interestingly, themes, which arose in the interviews, were similar between individuals *within* each group. Beyond that, the themes were nearly identical between the GP and patient groups.

### The positive impact of PA on pain

In general, both doctors and patients viewed PA as having a positive impact on people with CBP, not only with reducing pain but also for improving mood and overall health. In particular, the positive impact of outdoor PA was emphasised by interview partners:

*‘Even outside of work, I try to stay in motion and walk as much as possible. I’m aware that it’s really good for my back*.*’*

(Birgitt, patient [P], aged 51 years)

*‘Or “forest bathing” is quite new and popular at the moment. I let them know that simply going for a 30-minute walk on some soft forest floor is also good for your back.’* (Rolf, GP, aged 53 years) (*‘This Japanese practice* [of forest bathing] *is a process of relaxation; known in Japan as shinrin yoku. The simple method of being calm and quiet amongst the trees, observing nature around you whilst breathing deeply can help both adults and children de-stress and boost health and wellbeing in a natural way.’ *)^23^

Negative effects of PA, in terms of pain increase when performing unsuitable PA, were rarely verbalised by patients but did occur:

*‘I went to a “Tae Bo” session once and I had pain. Jumping lots wasn’t that great for the back.’*

(Louisa, P, aged 39 years) (Tae Bo is an exercise system combining elements of aerobics and kickboxing).

### Barriers affecting participation in PA

Interview partners reported barriers affecting patient participation in regular PA. Those could be categorised into internal and external causes. A complete overview of barriers with corresponding interview passages can be seen in Supplementary Box S1. Especially important were the subthemes around maintaining PA as a routine: ‘bad habits’, listlessness/psychosomatics, regional availability, COVID-19 restrictions, and lack of time.

### Internal barriers

Many physicians and patients saw insufficient long-term adherence, in the sense of maintaining PA as a routine, as a main barrier. This was regarded as especially challenging in times when pain had already improved:

*‘On days free of pain, I often forget it* [doing exercises].*’*

(Birgitt, P, aged 51 years)

PA becomes a secondary focus in daily life:

*‘Most of the time it* [physical activity] *gets lost in the daily routine.’*

(Harald, P, aged 30 years)

This seemed to be the case particularly after an interruption of PA:

*‘… then when there is a longer break again. Then it’s out of sight, out of mind again.’*

(Barbara, P, aged 58 years)

Interviewed GPs highlighted specific characteristics of individuals as barriers to motivation for PA (‘bad habits’):

*‘There are those couch potatoes that won’t do much. They will tell me “Yeah, I work out, by going to the washing machine twice a day.” Not much left to do there.’*

(Melanie, GP, aged 61 years)

Furthermore, GPs saw listlessness and psychosomatic aspects as an internal barrier:

*‘*… *people are lacking drive, which in turn makes getting them off their sofas harder … Sometimes there’s also something like a depressive component.’*

(Anke, GP, aged 45 years)

Some patients expressed feeling stigmatised and frustrated:

*‘I would like to have more support and conversations because it’s a real ordeal. It’s like I am being treated like I was faking my ailments. I feel helpless when I’m not taken seriously enough.’*

(Anna, P, aged 42 years)

Few patients described anxiety and avoidance behaviour:

*‘I was scared after having surgery on my knee … it’s like you’re walking and a voice in your head tells you to walk slower and be more cautious. Slow down. That’s still in my head.’*

(Anastasia, P, age not supplied)

GPs also saw secondary disease gain as a cause of reduced activity and worsening pain:

*‘You can try doing whatever you want; it usually only gets worse. The pain increases because there’s a certain desire to retire or receive disability benefits.’*

(Karl, GP, aged 60 years)

Both patients and GPs reported difficulties due to comorbidities:

*‘It of course gets a whole lot more difficult with motivation when they have knee arthrosis or similar, like an additional comorbidity that restricts their movement.’*

(Lena, GP, aged 42 years)

#### External barriers

Insufficient regional availability was one major external barrier in recruiting patients for PA:

*‘There are plenty of possibilities to get exercise, for example, water gymnastics. But all of the programmes are usually overloaded and it’s really, really difficult to get in.’*

(Anna, P, aged 42 years)

Patients and GPs described COVID-19 restrictions as a factor aggravating this situation:

*‘I’ve already been back to school but then corona broke out. Everything was shut down and closed again.’*

(Hans, P, aged 44 years)

Another important limitation to performing regular PA was patients’ lack of time, for example, due to work and personal circumstances:

*‘Work takes up most of my life; I live in two different cities.’*

(Christian, P, aged 59 years)

Some patients reported that loneliness and/or a lack of support prevented them from PA:

*‘I’ve given up on having my husband join me on walks … I get the feeling that he doesn’t want any company; just wants some alone time.’*

(Susanne, P, aged 68 years)

Some of the GPs stated that they did not have enough time in their daily work routine for giving advice to patients on PA:

*‘The thing with giving counselling and recommendations is that I honestly feel like I’m working in a disaster area … it’s always like I’m playing Blitz chess for the entire day … I really don’t have the time left to explain anything to anyone.’*

(Lukas, GP, aged 42 years)

Some of the GPs said that they did not feel confident advising patients on PA:

*‘The only thing we usually demonstrate is the “step bed storage”* [a lying position to relax the back] *... I don’t even know what I’m supposed to show them.’*

(Anke, GP, aged 45 years)

### Strategies and ideas to increase long-term maintenance of PA

Beyond the barriers influencing engaging in PA, a theme from both doctor and patient interviews was how behaviour change towards undertaking more PA could be achieved. In this context, interview partners emphasised different strategies to increase long-term maintenance of PA. The theme of strategies to increase PA included the following subthemes: sustainability; enjoyment; easy start; group dynamics and social contacts; commitment; low threshold; autonomy; and self-efficacy.

First, effective methods to motivate individuals with CBP should support patients to maintain sufficient levels of PA:

*‘Yeah, an app is a good idea, that gives you an impulse over and over … In a way that my alarm also tells me “you have to get up now”, the app would ask me “have you done your back exercises today already?”’*

(Christian, P, aged 59 years)

Among strategies to increase PA, enjoyment was a central subtheme for both patients and GPs:

*‘At the end of the day, it’s all about conveying that there’s fun in movement. Easy as that.’*

(Christian, P, aged 59 years)

Moreover, according to patients and GPs, PA should be easy to carry out:

*‘I mainly recommend going for walks because I don’t think it’s overstraining. Even if you’re not athletic and don’t do sports, it still does a good job.’*

(Lena, GP, aged 42 years)

**Figure 2.**
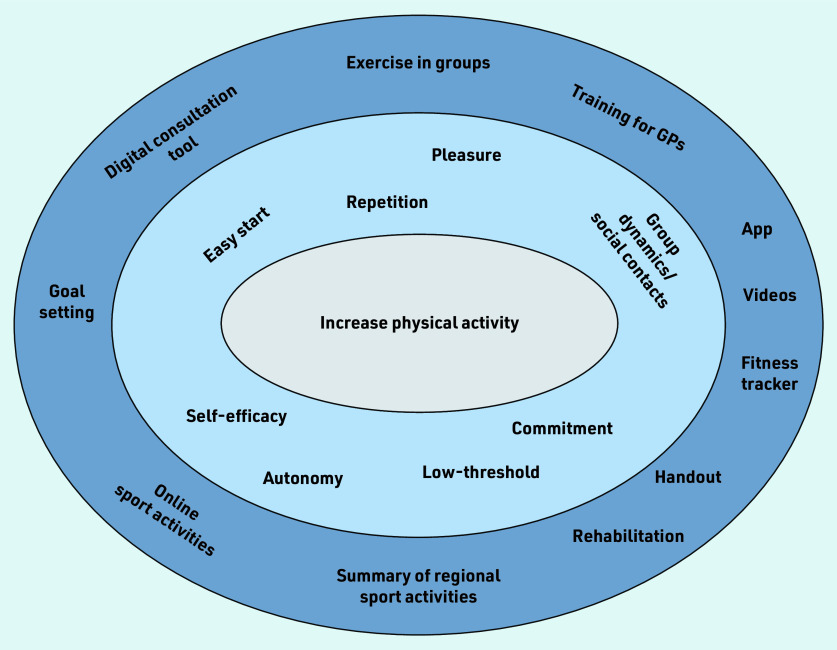
*Overview of motives (light blue) and concrete ideas (dark blue) to increase physical activity expressed by patients and GPs. A range of different motives and recommendations was stated. Most important was exercise in groups, providing a summary of regional sports activities, use of digital consultation tool, and providing a handout.*

Interview partners mentioned several concrete ideas to increase PA. Figure 2 provides an overview of these.

For many interview partners, performing PA in groups, namely in the form of exercise groups or individual meetings, was considered helpful:

*‘It’s definitely easier for me when I have company. Let’s look at Nordic walking* [enhanced walking technique using poles to work your upper body as well as your legs], *for example. I do that twice a week but never go by myself … and rehabilitation sport* [sport rehabilitators assist people with pain, injury, or illness involving the musculoskeletal system], *is a group sport by default, which is really, really great.’*

(Barbara, P, aged 58 years)

Furthermore, the possibility of providing a summary of regional sports activities was stated as useful to advise patients:

*‘I would appreciate more precise recommendations on sports programmes from my doctors … Just specific things I could do, that aren’t far from my home instead of “you could try this or that sport.”’*

(Louisa, P, aged 39 years)

Such a summary could be part of a handout or presented in a digital consultation tool, which were both considered advantageous:

*‘A website would make a lot of sense where you could download targeted content where you could then say, “Look, I would recommend doing this here in your case.”’*

(Melanie, GP, aged 61 years)

*‘In my conversations, a handout would make the most sense to show something to my patient.’*

(Matthias, GP, age not supplied)

Some GPs pointed out that specific exercise instructions they can give to their patients would be helpful:

*‘So what would actually make it easier is if you had a list or a plan with things that you could specifically advise people about, that you could maybe give them ... That there are concrete recommendations.’*

(Hannah, GP, aged 35 years)

Interestingly, goal setting and regular reminders, for example, in the form of an app, were controversial:

*‘I am careful not to be too controlling because I have no way of knowing how much truth there is to a patient’s answer.’*

(Riccardo, GP, aged 62 years)

*‘For me personally it would be good* [goal setting]*. Other people might feel too controlled, restricted or whatever. For me personally, it would have been good especially to upkeep motivation when I’m just getting started.’*

(Louisa, P, aged 39 years)

Interview partners were asked for their views on different options of pain visualisation. In particular, whether a certain form of visualisation was perceived as motivating. Views were diverse. Some preferred a simple visual analogue scale and even thought they were not being taken seriously when looking at a scale depicting smiling to unsmiling faces. Conversely, others expressed the advantages of a scale with smileys, for example, it being easy to understand, nice to look at, and inviting.

### The doctor–patient relationship

An important aspect influencing beliefs on PA and motivation was the doctor– patient relationship. Most patients and GPs expressed a satisfactory relationship. Some interview partners (GPs and patients) described contact between GPs and patients as mutualistic:

*‘It’s often like you have a treatment contract somewhere and as a doctor you obviously always have the wish to help your patient … It can be very hard sometimes because you have to be careful about how you communicate with the patient. You don’t want to make them feel like they are not understood or lectured from above.’*

(Carla, GP, aged 31 years)

*‘You always trust your doctor.’*

(Dmytro, P, aged 42 years)

There seemed to be a desire for a relationship based on partnership, especially by the doctors:

*‘Together with the patient, I make an effort to find opportunities to do sports that are also doable to them.’*

(Anke, GP, aged 45 years)

*‘I merely see myself as a supporter in the patient’s life plan.’*

(Matthias, GP, age not supplied)

Nonetheless, in some patients, this relationship has changed in a way that the patient themselves feels knowledgable on CBP. They tended to view themselves as consumers and doctors as service providers. Patients clearly define their needs and expect the GPs to provide them with information and prescriptions:

*‘That were the next steps and then — so to speak — I’ve just ordered what I want from my GP.’*

(Louisa, P, aged 39 years)

To some extent, this was also the perception of the GPs:

*‘Speaking from my own experience it’s more that the patients coming with back pain have an idea of pain-relieving shots. Some also ask for physiotherapy or even massage treatments.’*

(Carla, GP, aged 31 years)

However, some doctors view themselves as authority figures (in the sense of a paternal doctor–patient relationship) and expressed their frustration in the treatment of patients with CBP. They often believe patients do not follow their PA recommendations:

*‘The failure rate of our counselling concerning chronic back pain is definitely above 50%. As in, I recommend being more active and doing sports but the patients don’t comply.’*

(Rolf, GP, aged 53 years)

This might partly lead to a negative view of patients as *‘couch potatoes’* (Melanie, GP, aged 61 years) and *‘lazy’* (Karl, GP, aged 60 years).

In line with the feeling of frustration of GPs, some patients indicated feeling stigmatised by their GPs and other health providers:

*‘I would like people to be taken more seriously.’*

(Anna, P, aged 42 years)

When placing perceived doctor–patient relationships in connection with participant characteristics in the sample, older GPs (aged ≥60 years) tended to view their role in a paternalistic model and younger GPs (aged <60 years) in a partnership-based model.

## DISCUSSION

### Summary

This study explored the perceptions, barriers, and motivators of PA in individuals with CBP and GPs advising them. One main result was that the themes were very similar between patients and GPs.

The main barriers were maintaining PA as a routine, particularly when pain had already improved; insufficient regional availability, aggravated by COVID-19 restrictions; and lack of time. Furthermore, GPs highlighted specific characteristics of patients as barriers to having motivation for PA.

Outdoor PA was seen as having a particularly positive impact on physiological and mental health.

Interview partners mentioned a broad range of strategies and concrete ideas to increase PA, such as exercising in groups or using digital tools.

Both groups perceived their doctor– patient relationships as positive. However, the results highlighted some important differences: some GPs viewed their role as a partnership-based or paternalistic type of doctor–patient relationship, while patients tended to view themselves, in relation to their GP, as a service consumer. This might have led some GPs to develop a negative view of the patient characterised by aspects such as them having a sense of entitlement, and may be one reason for feelings of failure, frustration, and resignation on both sides.

### Strengths and limitations

An important strength of this study is that GPs and patients were interviewed in parallel; therefore, the researchers were able to rely on the views of the other interviewees when conducting and analysing the study. However, as ‘paired interviews’ were not conducted, the different views could not be directly contrasted. Another strength is that the study team consisted of researchers with different backgrounds, including medical, psychological, and biological. Study results were continuously discussed and further developed within the team. Therefore, results were double-checked against different views.

Some limitations have to be taken into consideration. Most of the interviewed patients were recruited by their GP. This, and the financial support provided by the study group, may have led them to take positions that they found acceptable to their GP and the study team. However, some participants clearly felt free to criticise. Further, all interview partners knew that views on PA would be discussed, which could have potentially resulted in a selection bias, as individuals who agreed to participate might have been more interested and more motivated.

Most of the interviews were performed via telephone because of COVID-19 restrictions. It is difficult to assess whether this had an impact on interviews.^24^ There is the possibility that it may have been more difficult to establish a connection with the interviewee than in a face-to-face interview; however, it is also possible that interviewees may have felt more relaxed talking on the telephone and more able to discuss sensitive topics. Interviews were held and coded by the first and second authors, both working as GPs. Personal experience in the field may have influenced study results.

Three researchers work as GPs, which might have influenced data collection and analysis. The GPs interviewed might have been more willing to share their experiences, while the patients might have been more reluctant or careful in their wording compared to a neutral interviewer. Personal experiences with PA and patients with CBP might have influenced the data analysis. To be able to interview patients and GPs from various backgrounds, the researchers extended recruitment beyond their local research practice network.

### Comparison with existing literature

The behaviour change wheel framework was used to understand views of PA.^25^ Interview partners expressed barriers in all three behavioural domains:

- capability to perform PA was restricted, for example, due to comorbidities;
- opportunity was described as limited, for example, due to COVID-19 restrictions; and
- motivation, a main barrier, as interviewees described long-term adherence (maintaining PA as a routine) as particularly difficult.

Looking at the intervention functions, interview partners expressed a broad range of strategies to increase PA. One main theme in this context was that the type of PA was unimportant, but that enjoyment was associated with PA. This is in line with the results of the Cochrane Review^15^ on adherence to exercise: similar to the present findings, Jordan *et al* have shown that it is not a specific form of exercise that leads to improved adherence and that patient preferences should therefore be taken into account.^15^ Furthermore, in previous randomised controlled trials (RCTs) it has been shown that goal setting and regular reminders can be effective to improve adherence.^15^^,^^26^ However, the present interview study revealed a broad range of views: some participants found goal setting and regular reminders supportive, whereas others expressed a strong opinion against those strategies.

The interviews in the present study revealed a discrepancy in the doctor– patient relationship: GPs saw their role in a partnership-based or paternalistic type, while patients partly faced their doctor with the idea of being a consumer.^27^ Allegretti *et al* have also described a mismatch of different models but not in the doctor–patient relationship model, rather in the explanation of the cause of back pain.^28^ In their qualitative study, physicians explained disease with a biopsychosocial model, whereas patients were interpreting back pain in the sense of a biomechanical model. In line with the present findings, this mismatch of different models also leads to a high level of frustration in physicians when caring for patients. Doctors felt relegated to a supporting role, which corresponds to the consumer model in the presented study sample. Despite the mismatch, the majority of interviewees in Allegretti *et al*’s study stated — as in in the present study — that they have a high level of trust in their GPs.^28^

The difference in the perceived doctor– patient relationship might lead to negative emotions on both sides. On the one side, in the current study, some patients mentioned feeling stigmatised and not taken seriously. On the other side, some GPs felt frustrated and seemed to have a partly negative view of patients with CBP. In line with the present results, a metasynthesis on patients’ experience of CBP noted a tense relationship with health professionals as one main theme. In some included studies patients felt stigmatised and viewed as culpable, seeking secondary gain, and being lazy.^29^

The discrepancy in perceptions of the doctor–patient relationship and resulting negative emotions is highly important as the relationship plays a key role in advising patients with CBP. Holt *et al* have demonstrated that the doctor–patient relationship is crucial for reassurance during low CBP consultations.^3^

Notably, patients and GPs indicated a lack of knowledge in consultation on PA and some GPs expressed not feeling confident advising patients on PA. This is in line with the results of Chatterjee *et al*, where in their sample more than half of the GPs did not feel confident in talking about exercise with patients.^11^ The present study revealed that a simple handout with local PA opportunities would have been considered helpful to overcome this lack of knowledge. Such support is also recommended by public health information, for example, Arthritis Research UK, the Department of Health and Social Care, NHS England, and Public Health England recommend leaflets for people with musculoskeletal conditions to provide PA interventions.^30^^,^^31^

### Implications for research and practice

To the best of the authors’ knowledge, this is the first qualitative study exploring views and experience of PA in both individuals with CBP and the GPs advising them. This study provides an important insight into contextual factors when advising individuals with chronic pain to participate in PA. The doctor–patient relationship plays an important role in consultation of individuals with CBP and this study revealed the impact of issues in that relationship on PA consultations. It is highly important to develop studies investigating the effect of promoting behaviour change, especially PA, on the doctor–patient relationship. Primary care plays a key role in advising patients on PA, and a better understanding of factors impeding and promoting PA in patients with CBP can directly translate into improved patient care.
